# Comparison of Early Postoperative Outcomes after Total Gastrectomy and D2 Lymph Node Dissection with and without Splenectomy

**DOI:** 10.5005/jp-journals-10018-1274

**Published:** 2019-02-01

**Authors:** Volkan Oter, Tahsin Dalgic, Ilter Ozer, Kadri Colakoglu, Murat Cayci, Murat Ulas, Erdal B Bostanci, Akoglu Musa

**Affiliations:** 1 Department of Gastroenterological Surgery, School of Medicine, Sakarya University. Sakarya/Türkiye; 2 Department of Gastroenterological Surgery, Turkiye YuksekIhtisas Teaching and Research, Hospital, Ankara/Türkiye; 3 Department of Gastroenterological Surgery, School of Medicine, Eskişehir Osmangazi University. Sakarya/Türkiye; 4 Department of Gastroenterological Surgery, School of Medicine, Recep Tayyip Erdoyğan University. Rize/Türkiye; 5 Department of Gastroenterological Surgery, Şevket Yılmaz Teaching and Research, Hospital, Bursa/Türkiye

**Keywords:** Gastric cancer surgery, Postoperative outcomes, Splenectomy, Total gastrectomy.

## Abstract

**Background:**

A famous prognostic ingredient for gastric cancer is the lymph node metastasis. Previously in the therapy of gastric cancer, splenectomy was considered as a definitive part of lymph node dissection. Currently, preservation of the spleen is the accepted approach during total gastrectomy and routine splenectomy is abandoned. The aim of this study was to estimate the impression of splenectomy for D2 lymph node dissection with total gastrectomy.

**Methodology:**

Between February 1998 and January 2012, 1531 patients underwent gastric cancer surgery. Of these 257 patients, 205 patients underwent total gastrectomy with splenectomy, and the remaining 52 underwent a spleen-preserving total gastrectomy.

**Results:**

No statistical difference between these two groups in terms of age, gender, comorbidity, stage and American Society of Anesthesiologists score, surgical complications were detected. A significant difference was not seen in these groups with regard to postoperative mortality too.

**Conclusion:**

Early postoperative results were similar after TG ± splenectomy. Performing splenectomy did not increase the postoperative morbidity and mortality.

**How to cite this article:** Oter V, Dalgic T, Ozer I, Colakoglu K, Cayci M, Ulas M, Bostanci EB, Akoglu M. Comparison of Early Postoperative Outcomes after Total Gastrectomy and D2 Lymph Node Dissection with and without Splenectomy. Euroasian J Hepatogastroenterol, 2018;8(2):108-111.

## INTRODUCTION

A famous prognostic ingredient for gastric cancer is the lymph node metastasis. In the gastric cancer treatment, splenectomy was considered as a part of lymphadenec-tomy, and previous reports revealed better results for survival in patients with splenectomy.^1^ Especially for tumors situated in the proximal stomach, total gas-trectomy (TG) and splenectomy was considered to be a standard procedure because of the high frequency of Lymph Nodes (LN) metastasis to hilar nodes for proximal gastric tumors.^2^ On the contrary, some researchers suggested that splenectomy could be a cause of additional morbidity and mortality^3,4^ and also recent reports showed that splenectomy had no effect on survival for proximal gastric tumors.^5,6^ Currently, preservation of the spleen is the accepted approach during total gastrectomy, and routine splenectomy is not recommended.

The aim of this study was to estimate the impression of splenectomy for D2 lymph node dissection with total gastrectomy.

## METHODOLOGY

Between February 1998 and January 2012, 1531 patients treated for gastric cancer. Two hundred and ninety-six of them underwent TG with D2 lymphadenectomy. Because of the tumor invasion to the adjacent organs; distal pancreatectomy and splenectomy was performed in 39 patients, which were excluded from this study. Left behind 257 patients, total gastrectomy with simultaneous splenectomy was performed in 205 patients and the remaining 52 underwent a spleen-preserving total gas-trectomy. The clinical features, mortality and morbidity rates of the groups were compared.

The clinical specifications which could potentially affect early postoperative results included age (<70 vs. >70), gender, comorbid conditions, tumor stage, *American Society of Anesthesiologists* (ASA) score, additional organ extractions. Besides, these clinicopathological features impact on postoperative morbidity was assessed.

Splenectomy was considered as part of LND, and also it was not defined as an additional organ resection.

Staging of the patients was performed due to the 2018 TNM staging (8th edition) system of the American Joint Committee on Cancer.

Any formed complication in the first thirty postoperative days was defined as surgical morbidity. Complications such as an intraabdominal abscess or fluid collection wound infection, ascites or serous drainage, subileus or ileus, fewer, intraabdominal hemorrhage, anastomotic leakage, chylous ascites were defined as specific complications; venous thromboembolism, respiratory, cardiac and neurological problems were defined as non-specific complications.

### Statistical Analysis

All statistical analyses were conducted using the statistical program statistical package for the social sciences (SPSS) version 16 for Windows (SPSS, Chicago, IL, USA). Continuous variables were expressed as mean± standard deviation or median (minimum-maximum), where applicable. Mann-Whitney U-test was applied for comparing median values, and the mean differences were evaluated by student’s t-test. Nominal data were compared by Fisher’s exact test or Chi-square, where suitable and a p-value less than 0.05 was considered statistically significant.

**Table 1:** **Table 1:** Clinicopathological characteristics of the patients

		*Splenectomy*				*Total*			
		*No*		*Yes*					
		*n=52*		*n=205*		*n=257*		*P value*	
Age									
<70		42(20,7%)		161(79,3%)		203		P: 0.771	
≥70		10 (18,5%)		44 (81,5%)		54			
Gender									
Male		36 (18,8%)		155 (81,2%)		191		P: 0,347	
Female		16 (24,2%)		50 (75,8%)		66			
Comorbidity									
No		29(20%)		116(80%)		145		P: 0.885	
Yes		23(20,5%)		89 (79,5%)		112			
Tumor Stage									
I		9(23,7%)		29(76,3%)		38		P: 0,818	
II		11(20,4%)		43(79,6%)		54			
III		17(20,9%)		64(79,1%)		81			
IV		13(17,1%)		63(82,9%)		76			
ASA									
I		22(24,7%)		67(75,3%)		89		P: 0,299	
II		28(19,2%)		118 (80,8%)		146			
III		2 (9%)		20 (91%)		22			
Additional									
Organ		41(17,9%)		188(82,1)		229		P: 0,008	
resection		11(39,3%)		17(60,7)		28			
No									
Yes									

**Table 2:** **Table 2:** Complication rates of the patients with and without splenectomy.

				*Splenectomy*							
*Complication*				*No*		*Yes*		*Total*		*p value*	
		No		34		137		171		p > 0,05	
				(65,4%)		(66,8%)		(66,5%)			
		Yes		18		68		86		p > 0,05	
				(34,6%)		(33,2%)		(33,5%)			
Total				52		205		257		p > 0,05	
				(100%)		(100%)		(100%)			

**Table 3:** **Table 3:** Details of surgical complications

		*Splenectomy*			
*Complication*		*No*		*Yes*	
Wound infection		4		9	
Abscess/collection		3		17	
Serous drainage		1		1	
ileus		-		5	
Fewer		2		5	
Bleeding		2		7	
Leak		4		7	
Chylous ascites		-		1	
Nonspesific		5		11	

## RESULTS

Left behind 257 patients, total gastrectomy with simultaneous splenectomy was performed in 205 patients, and the remaining 52 underwent a spleen-preserving total gastrectomy. Clinicopathological features are shown in Table 1. No statistical difference between the groups was detected with regard to age, gender, comorbidity, stage and ASA score. Additional organ resections were more common in patients without splenectomy.

Postoperative complication rates were 33.2% for patients with splenectomy and 34.6% for those without splenectomy. No significant difference was detected between two groups regarding surgical complications (Table 2). Details of complications are included in Table 3.

Postoperative mortality was observed in 1 patient (1,9%) among the patients without splenectomy, and in 8 patients among those with splenectomy (3,9%). There was no statistically significant difference between these two groups (p > 0.05). The mean number of hospital stay time was similar in these two groups (13.4 ± 13.4 days and 12.9 ± 11.4 days in patients with and without splenectomy respectively; p > 0.05).

With univariate analysis, development of complications was not significantly affected by the included risk factors. However, although insignificant, postoperative complication rates were higher in ASA 3 patients and in the presence of more than one additional organ resection (Table 4).

**Table 4:** **Table 4:** Potential risk factors for postoperative complications

		*Complication*							
		*No*		*Yes*		*Total*			
		*n = 171*		*n = 86*		*n = 257*		*p-value*	
*Age*									
<70		138 (67.9%)		65 (32.1%)		203		p: 0,462	
≥70		33 (61.1%)		21 (38.9%)		54			
*Gender*									
Male		127 (66.5%)		64 (33.5%)		191		p: 0,710	
Female		44 (66.7%)		22 (33.3%)		66			
*Comorbidity*									
No		99 (68.3%)		46 (31.7%)		145		p: 0,593	
Yes		72 (64.3%)		40 (35.7%)		112			
*Tumor Stage*									
I		23 (60.5%)		15 (39.5%)		38		p > 0.150	
II		30 (55.6%)		24 (44.4%)		54			
III		59 (72.8%)		22 (27.2%)		81			
IV		52 (68.4%)		24 (31.6%)		76			
*ASA*									
I		59 (66.3%)		30 (33.7%)		89		p: 0,127	
II		102 (69.8%)		44 (30.2%)		146			
III		10 (45.5%)		12 (54.5%)		22			
*Additional*									
*Organ resection*									
Yok		153 (66,8%)		76 (33,2%)		229		P:0,395	
1		17 (68,0%)		8 (32,0%)		25			
>1		1 (33,3%)		2 (66,7%)		3			

## DISCUSSION

Splenectomy had been slated for extract the LN round the splenic hilum and the artery. In gastric cancer patients, previous literature recommended that the addition of splenectomy to gastrectomy improved survival than gastrectomy alone.^7^ However, recently, spleen preservation during LND was reported to decrease complications without any detrimental effect on overall survival.^8^ Numerous recent studies advocated that addition of splenectomy to gastrectomy did not improve survival after total gastrectomy, and also it might lead to severe complications.^9^ In a study by Goto and colleagues, the results of splenectomy in cases with Siewert type II tumor was evaluated and it was concluded that splenectomy could be omitted without decreased curability.^10^

Yu et al. showed that splenectomy did not improve survival in the presence of lymph nodes metastasis along the splenic artery or splenic hilum.^11^ According to these findings, it may be suggested that the addition of splenectomy to gastrectomy shouldn’t be performed for advanced gastric cancer patients. Currently, splenectomy cannot be performed as a routine procedure.

However, Kosuga et al reported patients who had a metastatic lymph node on splenic hilum were exactly cured by total gastrectomy with splenectomy. They suggested that splenectomy should be considered only for selected patients with localized tumors on the greater curvature or Borrmann type IV.^12^

We used to perform routine simultaneous splenectomy during total gastrectomy and D2 LND, however after recent reports, we abandoned routine splenectomy. Currently, splenectomy is performed for Borrmann type 4 tumors and greater curvature.

Several studies showed that splenectomy had not increased the morbidity and mortality.^13^ On the contrary, Yao et al. revealed that mortality rates were similar, but morbidity was lower in the spleen-preservation group.^14^ In this study, postoperative complication rates were 33,2% and 34,6% in patients with and without splenectomy, respectively. Mortality rates were also similar. Our results show that spleen-preserving radical gastrectomy results are not diminished morbidity and mortality in the postoperative period. In a previous study; splenectomy was not efficacious for patients with splenic hilum metastasis in long-term survival.^15^ Therefore, gastrectomy without splenectomy will be enforceable and sufficient for locally-advanced proximal tumors.

In this study, we also evaluated potential risk factors for complications after total gastrectomy and D2 LND. None of the included risk factors has significantly increased the risk of postoperative complications. This may be a result of the number of patients in this study. In ASA 3 patients complication rate reached 54,5%. Also, although statistically insignificant, complication rates after resection of more than one additional organ due to the tumoral invasion was higher. Complication rates were similar after gastrectomy alone and gastrectomy with one additional organ resection, respectively. However, in 2 of 3 patients who underwent gastrectomy with 2 or more organ resection, postoperative complications occurred. In a previous study evaluating the effects of multiorgan resection for gastric cancer, we classified additional organ resections as one and more than one additional organ resection. In that study, single additional organ resection did not increase complication rates. However, more than one organ resection significantly increased complication rates.^16^ Our results in the current study support this finding. Although the rate of extra organ resection was higher in the splenectomy group, complication rates of these groups were similar as there were only 3 patients with more than one additional organ resection.

Among the risk factors in this study, advanced age may be regarded as risk factors for postoperative complications. However, age did not emerge as a potential risk factor. Similarly, in a previous study that concerning the impression of advanced age to gastric surgery, postoperative morbidity was similar in younger than 70 years patients and elderly than 70 years patients groups results. In that study mortality rate was statistically higher in the elderly group.^17^ Similar morbidity and higher mortality rates may be explained by the poor condition of the patient after gastric surgery.

## CONCLUSION

In conclusion, early postoperative results were similar in groups of total gastrectomy and D2 LND with or without splenectomy. Avoiding splenectomy did not reduce the risk of postoperative morbidity and mortality.

## References

[1] Toge T, Kameda A, Kuroi K, Seto Y, Yamada H, Hattori T (1985). The role of the spleen in immunosuppression and the effects of splenectomy on prognosis in gastric cancer patients. Nihon Geka Gakkai Zasshi.

[2] Ikeguchi M, Kaibara N (2004). Lymph node metastasis at the splenic hilum in proximal gastric cancer. Am Surg.

[3] Bonenkamp JJ, Hermans J, Sasako M, van de Velde CJ, Wel-vaart K, Songun I (1999). Extended lymph-node dissection for gastric cancer. N Engl J Med.

[4] Wu CW, Chang IS, Lo SS, Hsieh MC, Chen JH, Lui WY (2006). Complications following D3 gastrectomy: post hoc analysis of a randomized trial. World journal of surgery.

[5] Fang WL, Huang KH, Wu CW, Chen JH, Lo SS, Hsieh MC (2012). Combined splenectomy does not improve survival in radical total gastrectomy for advanced gastric cardia cancer. Hepato-gastroenterology.

[6] Mori EG, Celis J, Ruiz E, Payet E, Berrospi F, Chavez I (2012). Impact of splenectomy and/or distal pancreatectomy in the prognosis of the proximal gastric cancer. Revista de gastroenterologia del Peru: organo official de la Sociedad de Gastroenterologia del Peru.

[7] Yamamoto M, Baba H, Kakeji Y, Endo K, Ikeda Y, Toh Y (2004). Postoperative morbidity/mortality and survival rates after total gastrectomy, with splenectomy/pancreaticosple- nectomy for patients with advanced gastric cancer. Hepato- gastroenterology.

[8] Brar SS, Seevaratnam R, Cardoso R, Law C, Helyer L, Coburn N (2012). A systematic review of spleen and pancreas preservation in extended lymphadenectomy for gastric cancer. Gastric Cancer.

[9] Kunisaki C, Makino H, Suwa H, Sato T, Oshima T, Nagano Y (2007). Impact of splenectomy in patients with gastric adeno-carcinoma of the cardia. Journal of Gastrointestinal Surgery.

[10] Goto H, Tokunaga M, Sugisawa N, Tanizawa Y, Bando E, Kawamura T (2013). Value of splenectomy in patients with Siewert type II adenocarcinoma of the esophagogastric junction. Gastric Cancer.

[11] Yu W, Choi GS, Chung HY (2006). Randomized clinical trial of sple-nectomyversus splenic preservation in patients with proximal gastric cancer. British Journal of Surgery.

[12] Kosuga T, Ichikawa D, Okamoto K, Komatsu S, Shiozaki A, Fujiwara H (2011). Survival benefits from splenic hilar lymph node dissection by splenectomy in gastric cancer patients: relative comparison of the benefits in subgroups of patients. Gastric cancer.

[13] Yang K, Chen XZ, Hu JK, Zhang B, Chen ZX, Chen JP (2009). Effectiveness and safety of splenectomy for gastric carcinoma: a meta-analysis. World journal of gastroenterology: WJG.

[14] Yao XX, Sah BK, Yan M, Chen MM, Zhu ZG (2011). Radical gastrec-tomy with combined splenectomy: unnecessary. Hepatogas-troenterology.

[15] Nashimoto A, Yabusaki H, Matsuki A (2012). The significance of splenectomy for advanced proximal gastric cancer. International journal of surgical oncology.

[16] Ozer I, Bostanci EB, Orug T, Ozogul YB, Ulas M, Ercan M (2009). Surgical outcomes and survival after multiorgan resection for locally advanced gastric cancer. The American Journal of Surgery.

[17] Ozer I, Bostanci EB, Koc U, Karaman K, Ercan M, Ulas M (2010). Surgical treatment for gastric cancer in Turkish patients over age 70: early postoperative results and risk factorsfor mortality. Langenbecks Arch Surg.

